# Circadian Rhythms of Skin Surface Lipids and Physiological Parameters in Healthy Chinese Women Reveals Circadian Changes in Skin Barrier Function

**DOI:** 10.3390/biology13121031

**Published:** 2024-12-10

**Authors:** Lanxing Lv, Xiaoxi Yan, Mingyue Zhou, Huaming He, Yan Jia

**Affiliations:** 1 Beijing Key Laboratory of Plant Resources Research and Development, School of Light industry Science and Engineering, Beijing Technology and Business University, Beijing 100048, China; lanxinglv@163.com (L.L.); yanxiaoxi2023@163.com (X.Y.); 18813023881@163.com (M.Z.); 2 Key Laboratory of Cosmetic of China National Light Industry, School of Light industry Science and Engineering, Beijing Technology and Business University, Beijing 100048, China; 3 International School of Cosmetics, Beijing Technology and Business University, Beijing 100048, China

**Keywords:** circadian rhythm, skin surface lipid, skin physiological parameters, skin barrier

## Abstract

Background: Circadian rhythms are driven by the biological clock, an endogenous oscillator that generates approximately 24 h cycles in mammals. The circadian regulation of the lipid metabolism plays a crucial role in overall metabolic health. An analysis of the correlation between the skin’s physiological parameters and skin lipids can provide a better insight into the rhythmic changes in skin condition. Objectives: The aim was to reveal how skin surface lipids (SSLs) participate in the regulation of circadian rhythms in the skin and the importance of the circadian oscillation of facial lipid molecules in maintaining epidermal homeostasis. Methods: Changes in SSLs were assessed using UPLC-QTOF-MS. The skin’s physiological parameters were quantified using non-invasive instruments. Multivariate data analysis was employed to evaluate the differences. Results: Both skin surface lipids and physiological parameters exhibited certain circadian variation patterns. Four major lipid classes (fatty acids, glycerophospholipids, prenol lipids, saccharolipids) exhibited circadian rhythmic trends, with seven lipid subclasses contributing most significantly to the overall patterns observed. Among the physiological parameters assessed, sebum secretion, transepidermal water loss, moisture measurement value, and skin surface temperature exhibited sinusoidal circadian rhythms. Further analysis revealed significant correlations between fatty acids and saccharolipids with moisture measurement values, and between glycerolipids and pH value. In addition, lipids closely associated with the barrier such as unsaturated fatty acids and ceramide chain lengths correlated significantly with moisture measurement values. Conclusions: Through correlation analysis, the study elucidates the influence of diurnal fluctuations in skin surface lipids on skin barrier function. These findings hold significant implications for understanding skin barrier impairment associated with circadian rhythm disruptions.

## Simple Summary

This study focused on young Chinese women to investigate the variations in skin surface lipids and physiological parameters, such as moisture and temperature, over a 24 h period. By collecting the skin surface lipids’ amplitudes and measuring the physiological parameters at seven time points within 24 h, we identified significant circadian rhythmic changes in four major lipid classes and seven lipid subclasses. Additionally, we revealed a significant correlation between key lipids associated with skin barrier function and physiological parameters. The findings underscore the crucial role of skin surface lipids’ circadian rhythms in maintaining skin barrier health, providing a scientific foundation for the development of targeted skincare solutions that align with the body’s natural biological rhythms.

## 1. Background

The circadian rhythm regulates physiological, psychological, and behavioral changes in organisms, operating as an endogenous process maintained by the organism itself and exhibiting oscillatory patterns of approximately 24 h. In mammals, the circadian rhythm system comprises a central oscillator and numerous interconnected peripheral oscillators. The central oscillator is located in the suprachiasmatic nucleus (SCN) of the hypothalamus [1], The SCN clock is synchronized daily by environmental cues, known as Zeitgebers (derived from the German term meaning “time givers”) [2]. Among these, light–dark cycles are the predominant Zeitgeber for the central oscillator. Photoreceptors in the retina detect light, converting it into electrochemical signals that are transmitted to the SCN via the retinohypothalamic tract. Neural and hormonal rhythms emanating from the SCN [3], alongside other Zeitgebers such as feeding, temperature, oxygen, and metabolite levels, help synchronize peripheral clocks across the body [4]. This coordination ensures the temporal alignment of molecular and physiological processes in peripheral tissues with the external environment. Peripheral clocks can communicate with each other and can also maintain circadian rhythms independently of the SCN [5]. Nearly all cells in the human body possess a fixed or specific self-sustaining clock. These molecular clocks operate through transcription–translation feedback loops involving core clock components such as CLOCK, BMAL1, PER, and CRY. They regulate gene expression in a tissue-specific manner. These molecular oscillators not only control rhythmic gene expression but also interact with metabolic and hormonal networks to ensure temporal coordination among tissues [6]. Disruptions in these feedback loops or inter-tissue communication can lead to systemic dysregulation, impairing metabolic flexibility [7]. Consequently, when the robustness of an organism’s molecular clock diminishes, the overall adaptability of the organism also declines.

The skin responds to ultraviolet radiation and pathogens in a circadian manner [8], and the robustness of the molecular clocks within the skin cells is closely linked to skin homeostasis. Lipids are essential metabolites in the skin, playing crucial roles in signal transduction, energy metabolism, and transmembrane transport [9]. Lipidomics is a large-scale analysis of lipid characteristics, offering transformative insights into skin biology. Using advanced mass spectrometry techniques, researchers can perform quantitative and qualitative analyses of hundreds of skin surface lipids (SSLs) [10], including ceramides, cholesterol, and fatty acids. Lipidomics also facilitates the identification of biomarkers for skin diseases, providing potential therapeutic targets and strategies for personalized treatment. The lipid composition of the stratum corneum and the lipids from the sebaceous glands together form a complex yet unique mixture of SSLs, with approximately 10% originating from the stratum corneum and 90% from the sebaceous glands [11]. The appropriate balance of SSLs, in terms of relative abundance, composition, molecular organization, and dynamic functions, is crucial for maintaining skin health [12]. Numerous lipidomic studies have shown that alterations in SSL characteristics and the overall lipid profile are linked to the etiology of common skin diseases, such as atopic dermatitis [13], psoriasis [14], and acne [15]. Under healthy conditions, many of the physiological attributes of human skin, such as moisture content, transepidermal water loss (TEWL), pH value, sebum levels, and temperature, exhibit periodic variations [16,17]. Changes in skin surface lipids (SSLs) can directly or indirectly affect these physiological parameters. Currently, the circadian rhythms of skin cell transcriptomes [8] and physical parameters have been reported [16,17]. However, due to the difficulty in recruiting volunteers and the complexity of lipid sample collection (this experiment required volunteers to have a high level of cooperation in order to collect lipid samples at seven time points over a 24 h period), the circadian rhythm of skin lipids in healthy women has not been studied.

This study focuses on the facial skin of healthy women aged 18–25. Using high-throughput UPLC-QTOF-MS technology and methods established in previous research by our team [15,18,19], we comprehensively analyzed the circadian rhythms of SSLs and six different physiological skin parameters—moisture measurement value (MMV), transepidermal water loss (TEWL), sebum secretion (SSE), skin surface temperature (SST), forehead temperature, and pH value over a 24 h period. We also conducted a correlation analysis between SSLs and skin physiological parameters, exploring the underlying causes of circadian rhythm changes in these parameters from a lipidomics perspective.

## 2. Materials and Methods

### 2.1. Subject and Environmental Control

**Figure 1:**
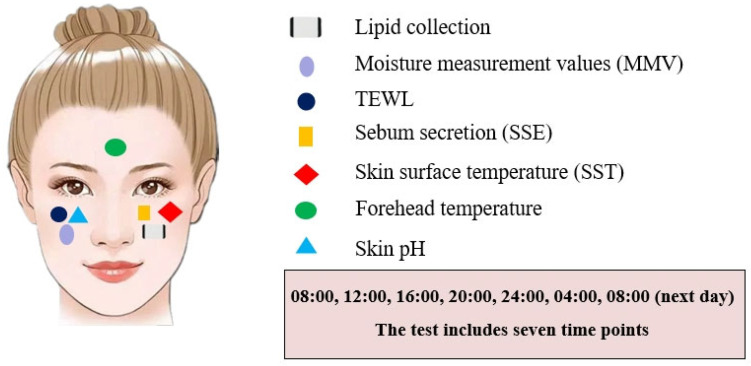
Lipid sampling and skin physiological-parameter-testing sites.

In order to observe the circadian rhythm trends of SSLs and their correlation with physiological parameters, a total of 35 young female volunteers (aged 23.1 ± 1.5 years) with healthy body condition and facial skin were recruited for the study after providing written informed consents. The study was conducted in accordance with the principles set forth in the Declaration of Helsinki (revised in 2000) and was approved by the Ethics Committee under approval number ERGZ20230517-05. All volunteers were not pregnant, breastfeeding, smoking, alcohol-drinking, not taking any medication for a minimum of last three months, and not menstruating. Lipid samples were collected from the same 35 volunteers at 4 h intervals (i.e., 08:00, 12:00, 16:00, 20:00, 24:00, 04:00, and 08:00 the next day) over a 24 h period, with simultaneous testing of the volunteers’ skin physiological parameters (Figure 1).

Environmental variables during the study were controlled, with a mean ambient air temperature of 25 °C and mean ambient air humidity of 45%. All volunteers arrived at the laboratory at 7:10, followed by face cleansing, and acclimatization for at least 30 min before the first sampling at 8:00. Volunteers were sampled and tested successively at a rate of 4 min per individual. After the first sampling, volunteers stayed in an environment with constant temperature and humidity (25 °C, 45%) until 11:10, at which point the volunteers cleansed their faces. Subsequently, thirty minutes later, the sampling and testing was repeated following the exact same order as the first time. This process was repeated at a total of seven time points. During the study period, volunteers maintained normal water intake, diet, and sleep patterns, and were allowed to engage in non-strenuous activities such as reading and writing.

### 2.2. Sample Collection and Preparation

The Sebutape^®^-S100 tape (Cuderm Corporation, Dallas, TX, USA) was applied to the zygomatic bone of the volunteer’s left cheek by an experimenter wearing sterile gloves. The tape was left in place for three minutes, then removed and labeled in a sterile EP tube before being stored on dry ice. All collected samples were stored in a −80 °C freezer until subsequent extraction. SSLs on the tape were extracted using a modified Bligh and Dyer method. Meanwhile, another experimenter measured six physiological parameters on the skin adjacent to the tape sampling site: MMV was assessed using a Corneometer (CM825; Courage & Khazaka Electronic, Cologne, Germany), TEWL was measured using Tewameter (TM300; Courage & Khazaka Electronic, Cologne, Germany), SSE was measured with a Sebumeter (SM815; Courage & Khazaka Electronic, Cologne, Germany), SST was measured with an Omron (MC-872J; Courage & Khazaka Electronic, Cologne, Germany), forehead temperature was assessed with an Omron (MC-872J; Courage & Khazaka Electronic, Cologne, Germany), and skin surface pH was assessed using a Skin-pH-Meter (pH905; Courage & Khazaka Electronic, Cologne, Germany).

### 2.3. Ultra Performance Liquid Chromatography (UPLC) Analysis

The chromatographic apparatus used was a Waters ACQUITY UPLC I-Class (Waters Corporation, Milford, MA, USA). The same total amount of lipid was analyzed for each sample. The UPLC protocol followed that of a previous study [18], with the eluent outlet connected to a quadrupole time-of-flight mass spectrometry (QTOF-MS) device for detection and characterization.

### 2.4. Mass Spectrometry Analysis

High-resolution mass measurements were performed using a Waters Xevo G2-XS QTOF-MS (Waters Corporation, Milford, MA, USA) equipped with an electrospray ionization interface operating in positive ion mode. The QTOF-MS parameters in this study were consistent with those in a previous study [18].

### 2.5. Data Extraction, Multivariate Data, and Statistical Analysis

Data were extracted using Waters Progenesis QI 2.0 and Ezinfo 3.0 (Waters Corporation, Milford, MA, USA), and multivariate statistical analyses were performed in conjunction with principal component analysis (PCA). Subsequently, characteristic lipid components were identified using orthogonal partial least squares discriminant analysis (OPLS-DA) and compared with the LIPID MAPS structure database (LMSD). The relative average contents of each lipid type at the seven time points were calculated. Statistical analyses were conducted to determine the circadian arithmetic mean of each TEWL, MMV, pH, and skin temperature data point for each subject. Means and standard errors were calculated for each variable at each time point and normalized to determine the average rate of change over the day (volunteer’s test value at 7 sampling time points/mean of that test value at 7 time points = average rate of change in test value over 24 h). After normalizing the rate of change in SSLs and physiological parameters in this manner, the data were pooled on a 24 h basis. Scatter plots of various parameters and lipids were generated using Origin 8.5 (OriginLab) to analyze their variation trends. A sine function was applied for curve fitting, and a coefficient of determination (R^2^) > 0.75 was considered indicative of a good fit to the original data trend. One-way analysis of variance (ANOVA) was performed using SPSS Statistics 22 on the normalized SSLs and physiological parameter data to evaluate the significance of differences across the seven time points, yielding *p*-values. Correlation analysis was also conducted to obtain R^2^ and *p*-values. A statistical probability (*p*-value) < 0.05 was considered significant, with *** *p* < 0.001, ** *p* < 0.01, and * *p* < 0.05.

When the analysis results meet both conditions—*p* < 0.05 in one-way ANOVA and R^2^ > 0.75 in sine curve fitting—the variable is considered to exhibit significant circadian rhythmicity.

## 3. Results

### 3.1. Rhythmic Oscillations in Lipid Species in the Facial Skin of Young Women

**Figure 2:**
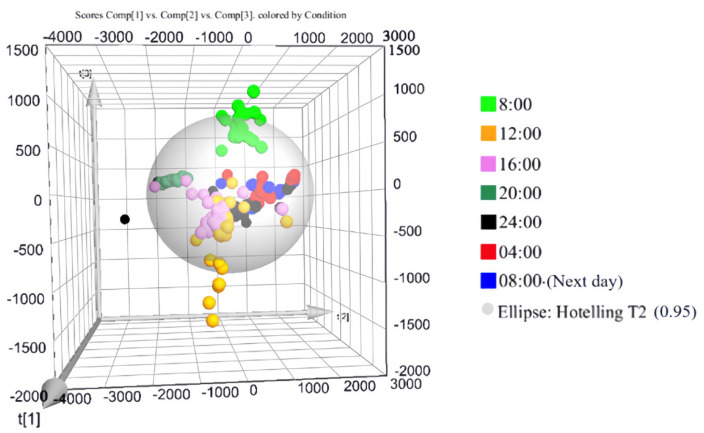
PCA plot of SSL samples collected at different time points. Each sphere represents a sample, with different colors indicating samples collected at different times.

The multivariate data were subjected to principal component analysis (PCA), and the results are presented in Figure 2. The seven sample groups demonstrated good within-group clustering and clear between-group separation, indicating substantial differences among the groups.

**Figure 3:**
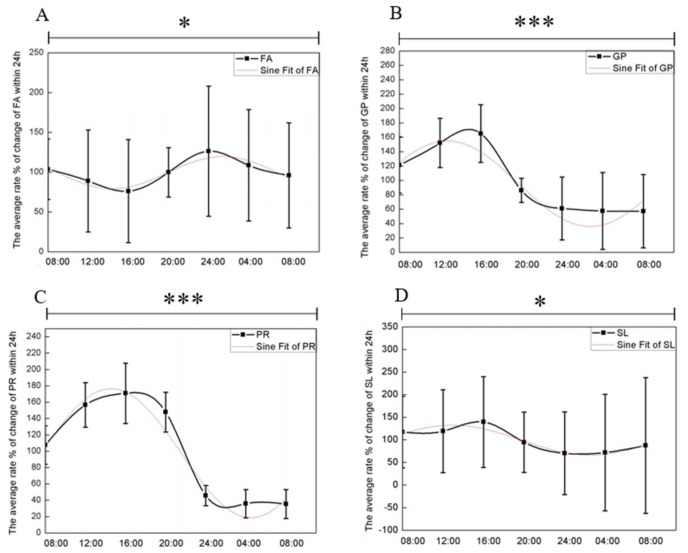
The four major lipid classes exhibit circadian rhythms, with a 24 h periodicity analyzed through one-way ANOVA and sine curve fitting (for further details, see Materials and Methods) (**A**) FA, *p* = 0.0376, R^2^ = 0.90619. (**B**) GP, *p* = 0.0001, R^2^ = 0.90898. (**C**) PR, *p* = 0.0001, R^2^ = 0.9436. (**D**) SL, *p* = 0.0466, R^2^ = 0.90287. *, significant differences at the *p* < 0.05 level; ***, significant differences at the *p* < 0.001 level.

Next, we analyzed the SSL composition at seven time points. A total of 1222 lipids were identified and classified into eight major classes following a search in the LIPIDMAPS database: fatty acids (FA), glycerolipids (GL), glycerophospholipids (GP), sphingolipids (SP), sterol lipids (ST), prenol lipids (PR), saccharolipids (SL), and polyketides (PK). Among these, four lipid classes FA, GP, PR, and SL exhibited significant circadian rhythmic variations in their average content over a 24 h period. The one-way ANOVA results for the four major lipid classes across seven time points demonstrated significant time-dependent differences. As illustrated in Figure 3, FA levels peaked around midnight and reached their lowest point around 16:00, displaying a negative sinusoidal trend. Conversely, GP, PR, and SL showed opposite trends, with levels decreasing near midnight and peaking in the afternoon, following a positive sinusoidal pattern.

**Figure 4:**
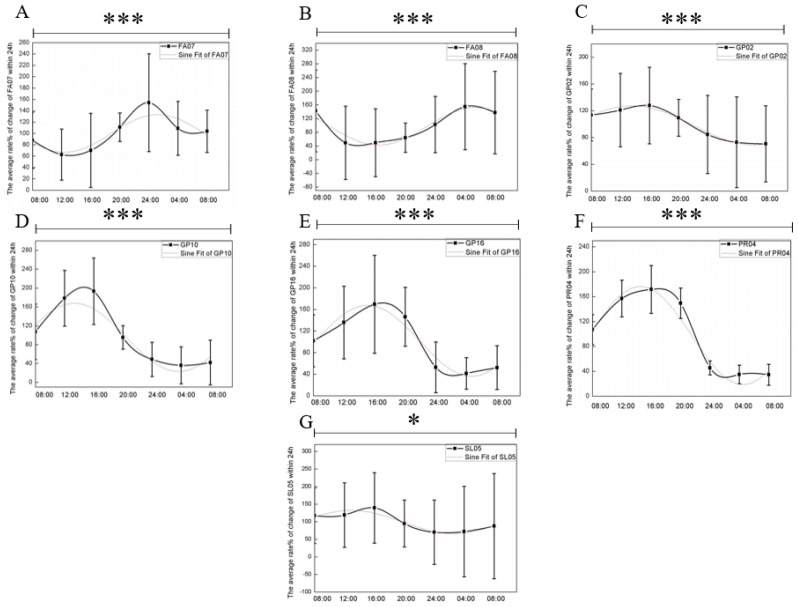
Circadian rhythmic changes in seven lipid subclasses over a 24 h period. (**A**) FA07, *p* = 0.0001, R^2^ = 0.85844. (**B**) FA08, *p* = 0.0001, R^2^ = 0.93566. (**C**) GP02, *p* = 0.0001, R^2^ = 0.98719. (**D**) GP10, *p* = 0.0001, R^2^ = 0.93067. (**E**) GP16, *p* = 0.0001, R^2^ = 0.9498. (**F**) PR04, *p* = 0.0001, R^2^ = 0.93452. (**G**) SL05, *p* = 0.0466, R^2^ = 0.77337. *, significant differences at the *p* < 0.05 level; ***, significant differences at the *p* < 0.001 level.

To determine which lipid subclasses contributed most to the circadian trends observed in the four major lipid classes (FA, GP, PR, SL), we conducted a further circadian rhythm analysis of their respective subclasses. The results of the one-way ANOVA demonstrated that seven lipid subclasses—fatty esters (FA07), fatty amides (FA08), glycerophosphoethanolamines (GP02), glycerophosphates (GP10), glycerophosphonocholines (GP16), hopanoids (PR04), and other acyl sugars (SL05)—showed significant time-dependent differences. As depicted in Figure 4, FA07 and FA08 exhibited trends consistent with their parent class FA, following a negative sinusoidal pattern after sine function fitting. Similarly, GP02, GP10 and GP16 mirrored the trends of their parent class GP. PR04 and SL05, also revealed sinusoidal trends consistent with their respective parent classes PR and SL.

### 3.2. Lipids Associated with the Skin Barrier Exhibit Circadian Rhythm Variations

**Figure 5:**
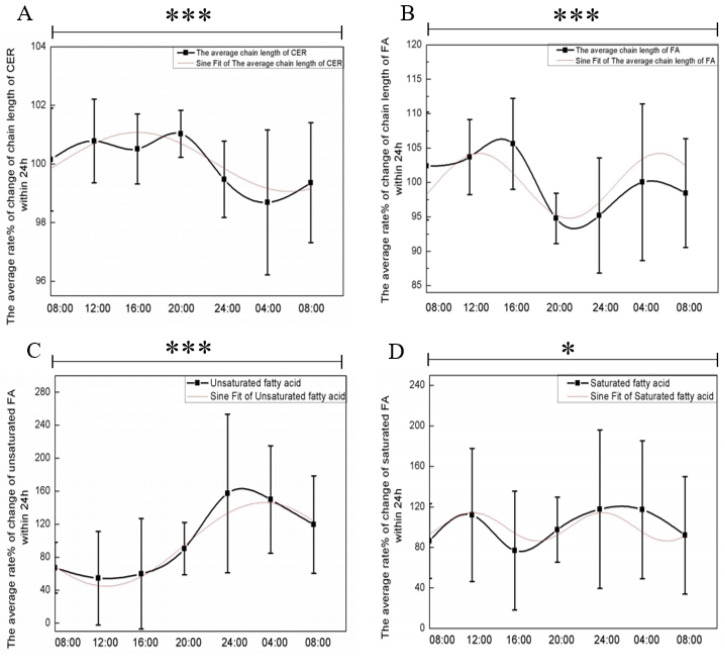
Circadian rhythmic changes in lipids associated with the skin barrier over a 24 h period. (**A**) The average chain length of ceramide, *p* = 0.0001, R^2^ = 0.75678, (**B**) the average chain length of fatty acids, *p* = 0.0001, R^2^ = 0.72738, (**C**) unsaturated fatty acids, *p* = 0.0001, R^2^ = 0.96164, (**D**) saturated fatty acids, 0.0193, R^2^ = 0.47159 over 24 h. *, significant differences at the *p* < 0.05 level; ***, significant differences at the *p* < 0.001 level.

It is well known that variations in the chain length of ceramides and fatty acids profoundly affect the skin barrier. This study also investigated the average rate of change in ceramide and fatty acid chain lengths in the skin over a 24 h period. The results of the one-way ANOVA demonstrated variations in ceramide chain length and fatty acid chain length, along with those in saturated and unsaturated fatty acids, exhibiting significant time-dependent differences. As shown in Figure 5, ceramide chain length peaked around 16:00 and reached its lowest point around 04:00, following a sinusoidal trend after sine function fitting. However, fatty acid chain length did not exhibit a clear 24 h circadian rhythm, with peaks observed at 16:00 and 04:00.

The circadian rhythm analysis of unsaturated fatty acids is shown in Figure 5C, where unsaturated fatty acids began to decline at 08:00, reaching a trough around 16:00, and peaking at 04:00, with a negative sinusoidal trend in the sinusoidal function fit, whereas saturated fatty acids showed no significant cyclic rhythmic trend (Figure 5D).

#### 3.2.1. Circadian Rhythms of Physiological Parameters in Healthy Female Facial Skin

**Figure 6:**
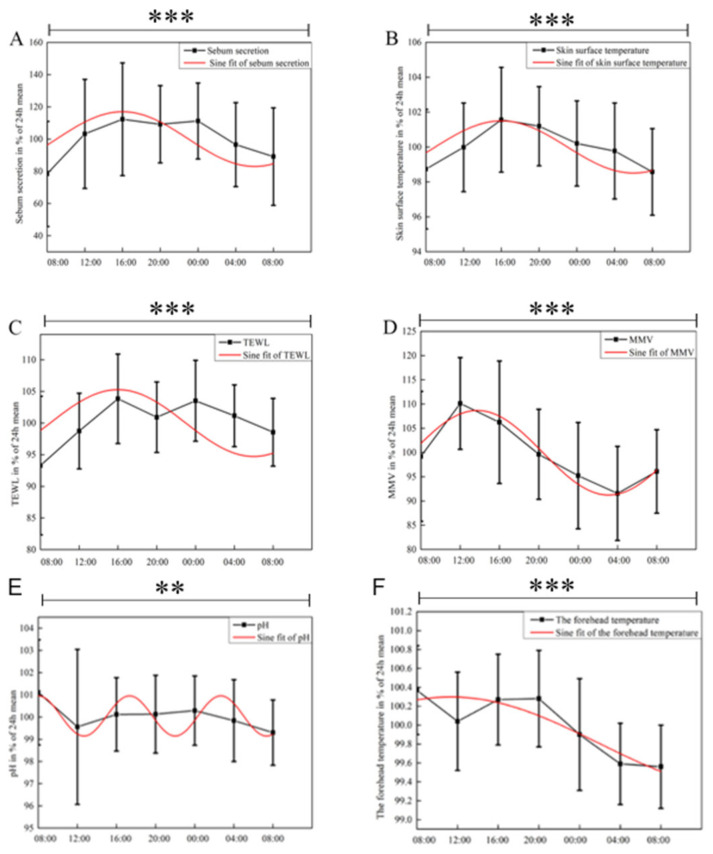
Circadian rhythmic changes in physiological parameters over a 24 h period. (**A**) SSE, *p* = 0.0001, R^2^ = 0.97377. (**B**) SST, *p* = 0.0001, R^2^ = 0.91158. (**C**) TEWL, *p* = 0.0001, R^2^ = 0.89372. (**D**) MMV, *p* = 0.0001, R^2^ = 0.88918. (**E**) pH, *p* = 0.0052, R^2^ = 0.55484. (**F**) Forehead temperature, *p* = 0.0001, R^2^ = 0.48089 over 24 h. **, significant differences at the *p* < 0.01 level. ***, significant differences at the *p* < 0.001 level.

This study characterized the facial physiological parameters of healthy Chinese women. After normalizing the data, the circadian rhythms of SSE, SST, TEWL, MMV, pH, and forehead temperature were analyzed. One-way ANOVA revealed significant time-dependent differences in these parameters. As shown in Figure 6, significant circadian rhythmic variations were observed in the SSE, SST, TEWL, and MMV values of the cheeks over a 24 h period, with all four parameters exhibiting sinusoidal variation trends. The SSE, SST, and TEWL values exhibited a gradual increase from 08:00, reaching a peak at 16:00, and subsequently declining until reaching a trough at 04:00 the following day. The rate of change in skin MMV, which was fitted by a sine function, demonstrated a gradual increase from 08:00, reaching a peak at approximately 14:00, followed by a decline and reaching a trough at 04:00. No significant rhythmic trends were observed in the skin surface pH and forehead temperature over the 24 h period.

#### 3.2.2. Correlation Analysis Between Surface Lipids and Physiological Parameters

**Figure 7:**
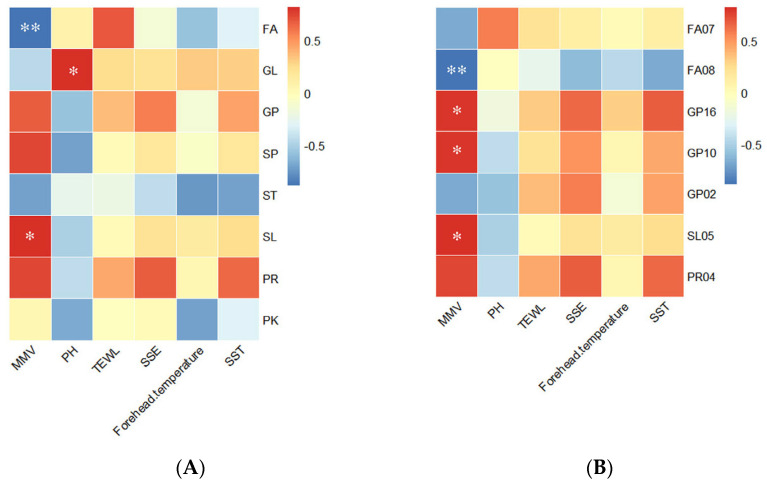
Heatmap of correlation analysis of skin surface physiological parameters with (**A**) eight lipid classes and (**B**) lipid subclasses. *, Significant correlation at the *p* < 0.05 level (two-tailed); **, significant correlation at the *p* < 0.01 level (two-tailed).

Lipid molecules are fundamental contributors to changes in skin condition, while physiological parameters serve as indicators of alterations in skin status. By analyzing the correlation between lipids and physiological parameters, we can identify the specific lipid classes that most significantly influence the circadian variations of these parameters. After normalizing skin physiological parameters and the eight lipid classes, we calculated the average values at seven time points and collected six sets of skin physiological parameters as well as eight sets of lipid data by Spearman correlation analysis (Figure 7A). The results revealed a negative correlation between MMV and the average relative content of FA (R^2^ = −0.893, *p* = 0.007), and a positive correlation between MMV and SL (R^2^ = 0.821, *p* = 0.023), as well as between pH and GL (R^2^ = 0.821, *p* = 0.023).

In order to explore the association of lipids undergoing rhythmic changes with physiological parameters, we correlated the seven subclasses of lipids that showed significant rhythmic oscillations in the above results with skin physiological parameters (Figure 7B). The results revealed that MMV was negatively correlated with FA08 (R^2^ = −0.893, *p* = 0.07) and positively correlated with GP16 (R^2^ = 0.786, *p* = 0.036), GP10 (R^2^ = 0.786, *p* = 0.036), and SL05 (R^2^ = 0.821, *p* = 0.023).

The rhythmic trends in MMV, fatty amide (FA08), glycerophosphate (GP10), and acylglucose (SL05) are shown in Figure 4 and Figure 6D. The trends in MMV (Figure 6D) and FA08 (Figure 4B) over a 24 h period were in a reciprocal trend. MMV and GP10 and GP16 (Figure 4D,E) showed the same trend, increasing with time, reaching a peak at 12:00 a.m. and then decreasing, reaching a trough at 04:00.

### 3.3. Correlation Between Skin Barrier-Related Lipids and Physiological Parameters

**Figure 8:**
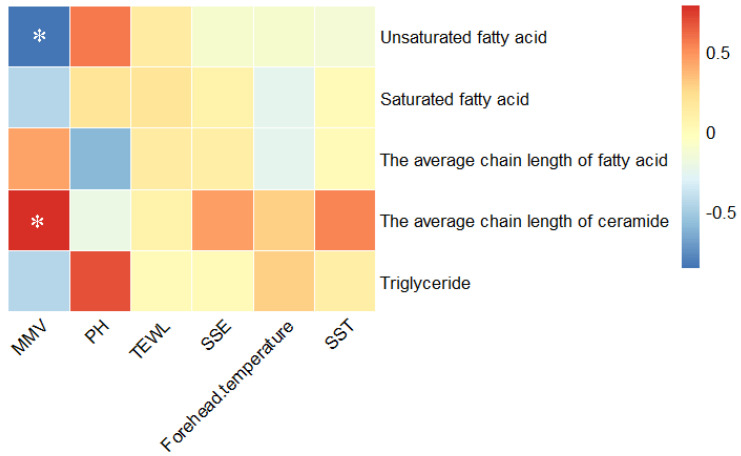
Heatmap of correlation analysis between skin physiological parameters and barrier-related lipids. *, significant differences at the *p* < 0.05 level.

Research has shown that epidermal lipids, such as ceramides, fatty acids (FAs), triglycerides (TG), and cholesterol, are key components in maintaining the epidermal permeability barrier (EPB). Abnormalities in EPB composition can lead to increased skin permeability and elevated transepidermal water loss (TEWL) [20,21,22]. Many skin disorders, including atopic dermatitis (AD), and acne, are characterized by defects or weakening of the epidermal barrier function. Berdyshev et al. [23] reported that children at high risk for AD exhibited elevated levels of unsaturated sphingolipids and short-chain ceramides. He Cong Wang et al. [24] also found that short-chain ceramides were significantly higher in AD patients compared to healthy controls, whereas long-chain ceramides were significantly lower. Research by Hui Bin Yin et al. [13] showed that TG levels in adults with AD were lower than in healthy individuals, and this reduction was associated with disease severity. Obumneme Emeka Okoro et al. [25] observed that in adolescents with acne, the abundance of TG, monounsaturated fatty acids (MUFA), and saturated fatty acids (SFA) in the skin surface lipids (SSLs) was significantly higher compared to healthy controls. We analyzed the correlation between these barrier-related lipids and skin physiological parameters. As depicted in Figure 8, MMV was negatively correlated with the average relative content of unsaturated fatty acids (R^2^ = −0.857, *p* = 0.014) and positively correlated with ceramide chain length (R^2^ = 0.786, *p* = 0.036). However, no significant correlations were found between physiological parameters and fatty acid chain length, saturated fatty acids, or triglycerides.

We normalized the average relative content of unsaturated fatty acids and skin MMV, obtaining the mean values for seven time points over 24 h. The circadian rhythm variation trends are shown in Figure 3A and Figure 5C. The changes in both components over the 24 h period followed an inverse pattern. After applying the same normalization process to the data of ceramide chain length and skin MMV, the circadian rhythm variation trends are shown in Figure 3A and Figure 5A. We found that the changes in ceramide chain length and MMV over time followed the same trend. This result further supports the positive correlation between ceramide chain length and skin MMV, with the ceramide chain length positively influencing the changes in skin MMV over time.

## 4. Discussion

An increasing body of evidence indicates that the molecular clock differentially regulates lipid metabolism across various organs and tissues. The autonomous biological clock system in the intestine modulates the digestion, absorption, and transport of lipids in a circadian manner [26,27]. Bile acids, which are critical components of bile, play an essential role in the digestion and subsequent absorption of food in the intestines, thus initiating the systemic lipid metabolism. Bile emulsifies and breaks down hydrophobic lipids, with studies indicating that bile acid production is rigorously controlled by the liver in a circadian manner [28,29]. A study by Ntsiki M. et al. [30] demonstrated that lipid content and composition in human skeletal muscle also exhibit circadian oscillations. Such changes may affect the muscle energy metabolism by regulating the size and number of lipid droplets. Circadian rhythms in lipid species may further influence fatty acid oxidation and insulin signaling, thus playing a key role in metabolic health. Circadian rhythms in the skin are intimately linked to immune processes and the maintenance of skin homeostasis. Epidemiological studies have associated shift work with an increased likelihood of developing psoriasis [31], as shift work disrupts the normal biological clock system. Inflammation resulting from circadian disruption could compromise skin integrity. Thomas et al. [32] found that the epidermal clock can integrate and subverts signals from the brain’s central clock. Our body and tissues exist within a constantly changing extracellular environment, and the brain’s central clock sends various signals in response to these dynamic conditions. However, the signals from the brain are not directly related to the tissues they affect. As a result, peripheral tissues selectively integrate or subvert these brain-derived signals to maintain the stability of their own physiological processes, such as the proper regulation of the cell cycle and DNA repair. This is crucial for the proper execution of daily physiological activities in the epidermis. Circadian oscillations of the key clock genes (e.g., PER, BMAL1) have also been observed in epidermal keratinocytes [33], melanocytes [34], and fibroblasts [35]. The biological clock mechanisms driving these oscillations influence a variety of physiological processes within these cells. Therefore, misalignment of circadian rhythms may disturb skin homeostasis.

In this study, we recruited 35 healthy Chinese women to characterize the circadian rhythms of skin lipids. Our findings indicated that four of the eight major lipid classes in the skin FA, GP, PR, and SL exhibited circadian variations in their content. Subsequent analysis of the subclasses within these lipid classes revealed that FA07, FA08, GP02, GP10, GP16, PR04, and SL05 displayed circadian trends consistent with their respective major lipid classes. Consequently, we hypothesize that these lipid subclasses contribute significantly to the circadian rhythm trends observed in their respective major lipid classes.

Ceramides, lipids unique to terrestrial mammals, are fundamental components of the stratum corneum that form a crucial permeability barrier. Alterations in the types and chain lengths of ceramide molecules are commonly observed in skin diseases related to the skin barrier [36]. A study of the ceramide profile in patients with atopic dermatitis (AD) found that the level of ω-O-N-acylsphingosine (EOS) in the skin of AD patients was significantly reduced [37]. Chisato Tawada et al. [38] analyzed ceramide profiles in the stratum corneum and observed that the total long-chain ceramide and fatty acid (FA) content in the skin of AD and psoriasis patients was significantly lower than that in healthy individuals. Variations in the chain length of fatty acids play a critical role in maintaining a healthy skin barrier. Very long-chain fatty acids (VLCFAs) and ultra-long-chain fatty acids (ULCFAs) are key components of ceramides, contributing to the rigidity and impermeability of the cell membrane in the skin [39]. These fatty acids are involved in the formation of the outer membrane layer, which provides chemical and mechanical strength to the skin. Therefore, changes in the composition and chain length of fatty acids can potentially alter membrane permeability. Short-chain fatty acids (SCFAs), secreted by skin microorganisms, have been shown to interact with PPARγ receptors, further regulating the synthesis of triglycerides in keratinocytes [40]. Our analysis revealed significant circadian rhythm changes in the chain length of ceramides in the skin of healthy women. Since ceramide biosynthesis primarily occurs in keratinocytes, we hypothesize that the biosynthesis and metabolic processes of ceramides in keratinocytes may be regulated by the molecular clock. However, the specific regulatory mechanisms remain to be explored. Additionally, while the chain length of fatty acids did not show significant periodic fluctuations, two peaks were observed at 16:00 and 04:00. The saturation of fatty acids exhibited periodic fluctuations. Given the essential role of fatty acids in regulating the integrity and function of the skin barrier, the mechanisms underlying the changes in their content and saturation require further investigation.

While characterizing the circadian rhythm of SSLs in healthy individuals, we also studied the physiological parameters of their facial skin. Among the six parameters analyzed, five—excluding pH and forehead temperature—exhibited clear circadian rhythms. The circadian trends of SSE and TEWL observed in our study, which exhibited peaks during the day and troughs at night, were consistent with the findings of Le Fur et al. [17]. In a study by M. Gloor et al. [41], ultraviolet (UV) exposure was applied to the backs of 19 subjects. Post-treatment lipid quantification tests revealed a significant increase in the total lipid content in the subjects’ backs. We hypothesize that UV exposure during the day may contribute to the peak of SSE in healthy individuals. Additionally, under UV irradiation, lipid components, including cholesterol and triglycerides, may undergo peroxidative reactions. These lipid peroxides can permeate the intercellular matrix, altering the bilayer structure and compromising the skin’s barrier integrity [16], which leads to elevated transepidermal water loss (TEWL). Therefore, we propose that UV radiation is a major external factor influencing sebum secretion and TEWL. In addition, we found that SST and SSE exhibited the same circadian rhythm patterns. Williams et al. [42] conducted a study where one side of the volunteers’ foreheads was kept at room temperature as a control, while the SST of the other side was manipulated by external conditions to study the effect of localized skin temperature changes on forehead SSE. The results showed a significant relationship between SST and SSE, leading us to hypothesize that variations in SST may influence facial lipid secretion. In our study, no significant circadian rhythm variation was observed in the facial pH of healthy Chinese women. Similarly, Le Fur et al. [17] reported no significant circadian variation in facial pH in healthy white women. However, Yosipovitch et al. [16] found a clear 24 h rhythm in facial pH in the Japanese population, with pH levels decreasing at night and increasing during the day. The facial pH was highest at noon (12:00) and lowest at midnight (24:00). We speculate that the discrepancies in these findings regarding facial pH could be attributed to ethnic differences, environmental factors, and variations in experimental conditions.

Our analysis of SSLs and physiological parameters revealed a negative correlation between MMV and the average relative content of fatty acids, as well as a positive correlation between MMV and the average relative content of sphingolipids (SL). In mammals, fatty acids (FA) are crucial for the structural function and physicochemical properties of cell membranes. For example, polyunsaturated fatty acids influence membrane permeability, membrane elasticity [43,44], membrane fusion, and vesicle formation [45,46]. From a biophysical perspective, an increase in polyunsaturated fatty acid content in phospholipid membranes also reduces their thickness and affects the geometric arrangement of lipids [47]. Cholesterol, a subclass of sphingolipids (SL), plays a key role in the development of the epidermal permeability barrier in the skin. It is a precursor for the synthesis of local steroid hormones and affects keratinocyte differentiation, corneocyte shedding, and barrier repair [48]. The evidence above suggests that fatty acids (FA) and cholesterol play a crucial role in the composition of cell membrane structure, membrane permeability, and the barrier function of cells. The causes of MMV are diverse, as various molecules within skin cells interact with each other to maintain skin homeostasis. Our correlation study between MMV and lipids aims to establish a connection between changes in lipid types and quantities and skin phenotype. Therefore, we hypothesize that changes in the types and quantities of these two lipid classes during their biosynthesis, conversion, and metabolism within the cell have a significant impact on the skin barrier function throughout the day. Furthermore, triglycerides (TG), a subclass of glycerolipids (GL), can be broken down by surface skin microorganisms to produce propionate, which can influence skin surface pH [49]. Our experimental results show a positive correlation between pH and the average relative content of GL. Thus, we speculate that GL plays an important role in regulating skin pH. In further subclass lipid analysis, we observed that the variations of MMV and fatty acyl amide (FA08) followed an inverse trend over the 24 h period, while MMV and glycerophosphates (GP10) exhibited a similar rhythmic trend. Our study is the first to identify a correlation between these two lipid subclasses and skin barrier-related physiological parameters. However, since no reports linking fatty acyl amide and glycerophosphates with skin barrier functions exist, their mechanisms warrant further investigation.

## 5. Conclusions

This study is the first to characterize the circadian rhythm variations of skin surface lipids (SSLs) in healthy individuals. Data analysis revealed significant circadian rhythm fluctuations in four major lipid classes—FA, GP, PR, and SL. Further subclass lipid analysis identified seven lipid subclasses—FA07, FA08, GP02, GP10, GP16, PR04, and SL05—that exhibited significant circadian rhythm changes. We further analyzed key lipids associated with skin barrier function and found significant circadian rhythm fluctuations in ceramide and FA chain lengths, as well as unsaturated fatty acid content. Additionally, based on existing reports, we extended our analysis to include the circadian rhythm variations of skin physiological parameters in Chinese women and correlated these parameters with lipid profiles. From a lipidomics perspective, we examined potential reasons for these circadian rhythm fluctuations. The primary function of SSLs is to form a natural protective barrier in collaboration with the facial stratum corneum and skin microbiota. SSL components are distributed between the skin surface and the stratum corneum in a specific ratio, maintaining skin health. Changes in total lipid content or individual components on the skin surface can affect skin barrier function. Therefore, we believe that studying SSLs in healthy individuals is crucial for understanding daily variations in skin barrier function. The circadian clock regulates the metabolism of various organs and tissues in our body. Both the central clock and peripheral clocks jointly control behavioral rhythms, and disruptions in these behavioral rhythms can, in turn, affect the entire circadian system. Irregular eating patterns, disrupted sleep cycles, and exposure to blue light from electronic devices in modern life may all contribute to circadian rhythm disturbances. Such disturbances can impair the normal barrier function of the skin. Therefore, we believe that research into the mechanisms linking circadian rhythms with various physiological processes and functions of the skin is both meaningful and necessary. This research could provide valuable insights for addressing skin issues caused by circadian rhythm disruptions and offer guidance for the development of related skincare products. Currently, this study focuses on the circadian rhythm of skin surface lipids in healthy Chinese women, but future research could expand to include subjects of different genders, regions, and ethnicities. Further exploration is needed to understand how changes in key lipids related to the skin barrier are regulated by the skin molecular clock.

## Data Availability

The datasets used and/or analyzed during the current study are available from the corresponding author on reasonable request.

## List of Abbreviations

UPLC/QTOF-MS: Ultra performance liquid chromatography/quadrupole time-of-flight mass, spectrometry and multivariate data analysis, SSL: skin surface lipid, SC: stratum corneum, SCN: suprachiasmatic nucleus, HPA: hypothalamic–pituitary–adrenal, TEWL: transepidermal water loss, MMV: moisture measurement value, SSE: sebum secretion, SST: skin surface temperature, PCA: principal component analysis, OPLS-DA: orthogonal partial least squares discriminant analysis, LMSD: LIPID MAPS structure database, FA: fatty acids, GL: glycerolipids, GP: glycerophospholipids, SP: sphingolipids, ST: sterol lipids, PR: prenol lipids, SL: saccharolipids, PK: polyketides, LCFAs: long-chain fatty acids, SCFAs: short-chain fatty acids, EPB: epidermal permeability barrier, SFA: saturated fatty acids, MUFA: monounsaturated fatty acids, UV: ultraviolet, ULCFAs: ultra-long-chain fatty acids, VLCFAs: very long-chain fatty acids, AD: atopic dermatitis.
